# Wearable Multi-Sensor Fusion for Field Assessment of Dynamic Postural Stability During the Curling Delivery in Young Adult Curlers: Integrating Trunk Kinematics, Plantar Pressure and Core-Muscle Activation

**DOI:** 10.3390/s26185928

**Published:** 2026-09-19

**Authors:** Bowen Zhang, Jiying Wu, Tianhao Han, Jian Zhang, Daqi Jiang

**Affiliations:** 1 Department of Physical Education, Northeastern University, Shenyang 110819, China; 2 School of Management and Journalism Communication, Shenyang Sport University, Shenyang 110819, China; 3 School of Mechanical Engineering & Automation, Northeastern University, Shenyang 110819, China; 4 National Frontiers Science Center for Industrial Intelligence and Systems Optimization, Northeastern University, Shenyang 110819, China

**Keywords:** athlete monitoring, curling, dynamic balance, inertial measurement unit, machine learning, plantar pressure, sensor fusion, surface electromyography, wearable sensors, young adult curlers

## Abstract

Postural control in curling is expressed during a dynamic, sport-specific delivery on a single sliding leg, yet it is still assessed almost exclusively with static single-leg force-plate protocols that poorly reflect on-ice demands. This study developed and validated a wearable multi-sensor system that fuses trunk and pelvis inertial measurement units (IMUs), instrumented plantar-pressure insoles and core-muscle surface electromyography (sEMG) to quantify dynamic postural stability during the simulated curling delivery in 110 young adult curlers (age range 18–24 years). A four-phase segmentation of the delivery was implemented, and a compact 28-feature set was extracted and combined through a complementary Kalman fusion scheme into a single Dynamic Stability Index (DSI). Validated against a reference force plate during static single-leg standing, the IMU-derived sway path showed a small bias (~0.4 cm) with 95% limits of agreement of approximately +4.4 to −3.6 cm, and between-day reliability of the fused DSI was excellent (ICC(3,1) = 0.90, 95% CI [0.82, 0.95]). A random-forest classifier discriminated high- from low-stability deliveries with 91.8% accuracy (95% CI [87.2%, 95.1%]; AUC = 0.92, 95% CI [0.87, 0.96]), outperforming any single-sensor or static force-plate baseline. Sensor-derived dynamic metrics correlated with external-oblique strength, trunk proprioceptive error and motor self-efficacy (Spearman ρ ranging from −0.55 to 0.51, all *p* < 0.001 after Holm–Bonferroni correction), and dynamic instability increased monotonically across descending self-efficacy tertiles. The proposed system provides a portable, context-relevant and interpretable tool for balance profiling in young curlers, and demonstrates that multi-sensor fusion substantially improves measurement fidelity over conventional single-modality assessment.

## 1. Introduction

Postural balance—the ability to control the projection of the centre of mass over a moving base of support—is a foundational determinant of athletic performance and injury prevention, and it is especially critical during the asynchronous physiological, sensory and cognitive development of adolescence and young adulthood [1,2]. Curling imposes an unusually demanding postural problem: the athlete slides from the hack on a single flexed leg across low-friction ice while simultaneously stabilising the trunk, tracking a distant target and releasing the stone with fine force modulation [3]. This closed, high-precision skill relies on sustained activation of the core stabilising muscles, among which the external oblique is a principal contributor to coronal-plane trunk control during unilateral support [4,5].

Despite the manifestly dynamic nature of the delivery, curling balance is still evaluated overwhelmingly through static single-leg standing on laboratory force plates [6]. Such protocols capture only a fraction of the sensorimotor demands actually encountered on the ice, because they remove the sliding base of support, the anticipatory trunk preparation and the fine release-phase force modulation that define the sport-specific task.

Balance is governed by the central integration of visual, proprioceptive and vestibular information, with sensory weights that are continuously and adaptively reallocated according to the reliability of each channel [7,8,9]. Proprioceptive fidelity determines the signal-to-noise ratio of the feedback loop and thereby the precision with which muscular force can be converted into stable posture [10,11,12]. Layered on top of these physiological and sensory mechanisms, psychological factors—particularly motor self-efficacy—shape attentional allocation and the efficiency of muscle mobilisation, and have been shown to predict performance most strongly in closed-skill sports such as curling [13,14,15]. A comprehensive balance assessment for curlers should therefore quantify the kinematic, kinetic and neuromuscular expression of stability during the actual delivery, rather than during an artificial static task.

### 1.1. Wearable Sensing of Postural Stability

The last decade has seen rapid progress in wearable and portable instrumentation for movement analysis. Body-worn inertial measurement units (IMUs) recover trunk sway with strong agreement against force-plate centre-of-pressure (CoP) metrics, offering an out-of-laboratory alternative that is inexpensive, unobtrusive and field-deployable [16,17,18]. Sensor-placement and signal-processing choices materially affect accuracy: mid-trunk and sacral placements are most informative, and band-pass filtering with task-appropriate cut-offs is required to isolate the postural signal [19,20,21]. In parallel, textile and capacitive plantar-pressure insoles now provide portable, dynamic CoP trajectories with acceptable reliability during locomotor and sport tasks [22,23,24]. Surface electromyography (sEMG) adds the neuromuscular dimension, capturing the timing and co-contraction of the core muscles that generate corrective trunk torque. Crucially, no single modality captures the full picture; kinematic, kinetic and neuromuscular sensors are complementary, and their principled fusion is expected to yield a more faithful description of dynamic stability than any modality alone. Complementing wearable sensing, vision-based motion analysis has also advanced rapidly; silhouette-based cross-view gait recognition using a multi-scale temporal difference unit demonstrates that dynamic spatiotemporal features can be effectively extracted from motion sequences for human movement assessment [25].

### 1.2. Machine Learning and Sensor Fusion for Balance

Multi-sensor data streams are naturally suited to machine-learning analysis. Support-vector machines and tree ensembles applied to inertial and pressure features have classified balance ability and fall risk with good accuracy, and multi-sensor combinations consistently outperform single-sensor inputs [26,27]. Beyond conventional classifiers, attention-enhanced deep learning architectures have demonstrated strong performance in real-time object tracking and industrial precision control, showing that attention mechanisms effectively prioritise task-relevant features in complex sensory data [28,29]. Recent work in wearable gait and balance assessment further demonstrates that ensemble models trained on fused inertial–pressure features achieve higher discriminative accuracy than single-modality models, particularly when feature-level fusion is combined with model-agnostic interpretability methods [30,31]. A recognised limitation of such models is their opacity; recent work therefore pairs classification with model-agnostic explainability, using approaches such as SHAP values and permutation importance to expose which sensor-derived features drive a decision, thereby making the output actionable for coaches and clinicians [30,31]. To date, however, these tools have been developed largely for clinical gait and fall-risk populations; their translation to the specialised, dynamic balance task of the curling delivery in developing athletes remains unexplored.

### 1.3. Aims and Hypotheses

The visual dependence of balance, the developmental immaturity of the proprioceptive system in adolescence and young adulthood, and the modulating role of self-efficacy all imply that a static, eyes-open force-plate score is an incomplete—and potentially misleading—index of a curler’s true stability [32,33,34]. The present study therefore designed a synchronised wearable multi-sensor system and evaluated it during a simulated curling delivery. We tested four hypotheses:

**H1.**  *IMU-derived sway metrics show acceptable agreement with a reference force plate (assessed during static single-leg standing) and acceptable between-day reliability, supporting the validity of field-based dynamic assessment*.

**H2.**  *A fused Dynamic Stability Index (DSI) that integrates trunk kinematics, plantar-pressure CoP and core-muscle co-contraction classifies high- versus low-stability deliveries more accurately than any single sensor or a static force-plate baseline*.

**H3.**  *Sensor-derived dynamic stability metrics are associated with external-oblique strength, trunk proprioceptive error and motor self-efficacy, and these associations are stronger for the fused DSI than for individual raw features*.

**H4.**  *Dynamic instability during the delivery increases across descending motor-self-efficacy tertiles, revealing a psychological gradient that static assessment obscures*.

## 2. Materials and Methods

### 2.1. Study Design

This was a cross-sectional instrument-validation and field-measurement study combining (i) development and calibration of a synchronised wearable multi-sensor system, (ii) criterion validation against a laboratory force plate, and (iii) cross-sectional measurement of dynamic postural stability during a simulated curling delivery. All procedures were carried out in a temperature-controlled laboratory equipped with a regulation curling hack and a 6 m synthetic sliding lane, allowing the delivery to be reproduced under standardised conditions.

### 2.2. Participants

A total of 110 curlers (62 male, 48 female; age 21.2 ± 2.3 years, range 18–24 years; height 174.3 ± 6.8 cm; body mass 68.5 ± 9.2 kg; curling-specific training experience 2.5 ± 1.1 years) were recruited from provincial professional sports schools and city-level amateur training teams. “Professional sports schools” refers to government-funded boarding institutions that provide both general secondary education and systematic, daily sport-specific training; “amateur training teams” refers to club-based after-school training programmes with 3–5 structured sessions per week. All participants were actively competing curlers at the time of testing, and the reported training experience refers specifically to systematic curling training.

Male participants (*n* = 62): age 21.5 ± 2.2 years, height 177.1 ± 5.4 cm, body mass 72.3 ± 7.8 kg, curling training 2.7 ± 1.0 years. Female participants (*n* = 48): age 20.8 ± 2.4 years, height 170.6 ± 5.9 cm, body mass 63.6 ± 6.5 kg, curling training 2.2 ± 1.2 years.

Inclusion required at least one year of systematic curling training and the absence of any trunk or lower-limb joint injury or neurological disorder in the preceding six months. No participant had interrupted training in the month before testing. All 110 recruited participants completed the testing protocol and were included in the primary analyses; no trials or participants were excluded due to technical failure, incomplete recording or illness. Written informed consent was obtained from all participants.

### 2.3. Wearable Multi-Sensor System

**Figure 1:**
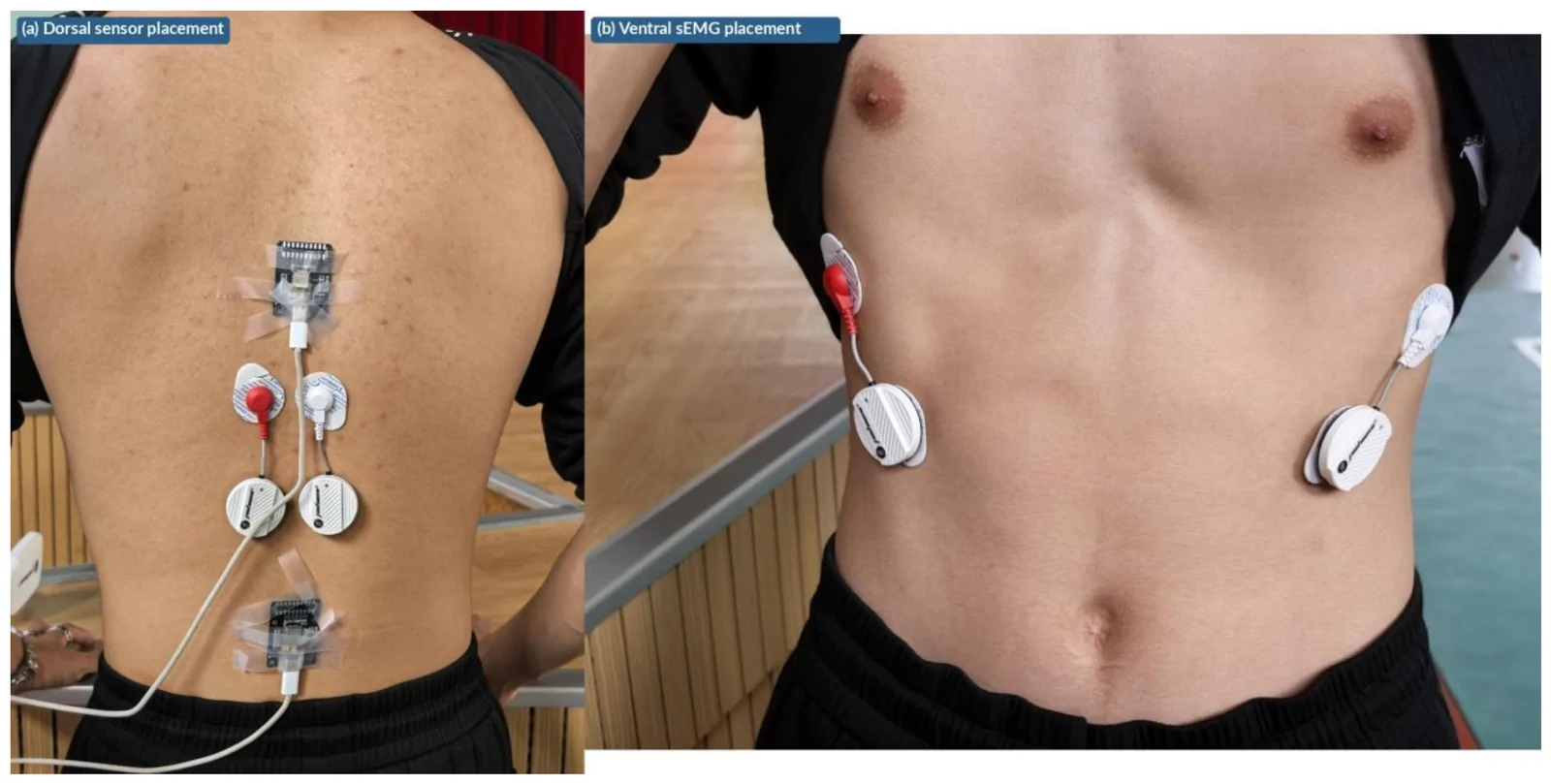
Wearable multi-sensor system: (**a**) dorsal placement of trunk (T3) and pelvic (S2) IMUs and bilateral erector spinae sEMG electrodes; (**b**) ventral placement of bilateral external oblique sEMG electrodes. Plantar-pressure insoles were worn inside both shoes (not visible).

The system comprised three synchronised modalities (Figure 1). Two nine-axis IMUs were mounted over the third thoracic vertebra (T3, upper trunk) and the second sacral level (S2, pelvis) using double-sided medical tape and elastic straps. These sites were selected because upper-trunk and sacral placements provide the most reliable estimates of postural sway during dynamic tasks, capturing both thoracic and lumbopelvic control [19,20,21]. Although the IMUs incorporate a magnetometer, magnetometer data were not used owing to substantial magnetic interference from the metal lane frame and force-plate housing; only accelerometer and gyroscope signals contributed to orientation estimation. A pair of capacitive plantar-pressure insoles recorded bilateral plantar-pressure distribution and in-shoe centre of pressure, and bipolar sEMG electrodes were placed on the delivery-side external oblique and erector spinae following SENIAM guidelines.

Before each session, sensors were calibrated per manufacturer protocol: IMUs were placed on a level surface for gyroscope bias and gravity-vector estimation; pressure insoles were zeroed under no load and calibrated against the participant’s body weight; and sEMG electrode impedance was verified (<10 kΩ) followed by a maximal voluntary contraction trial for amplitude normalisation. Detailed specifications are listed in Table 1.

### 2.4. Delivery Task and Protocol

**Figure 2:**
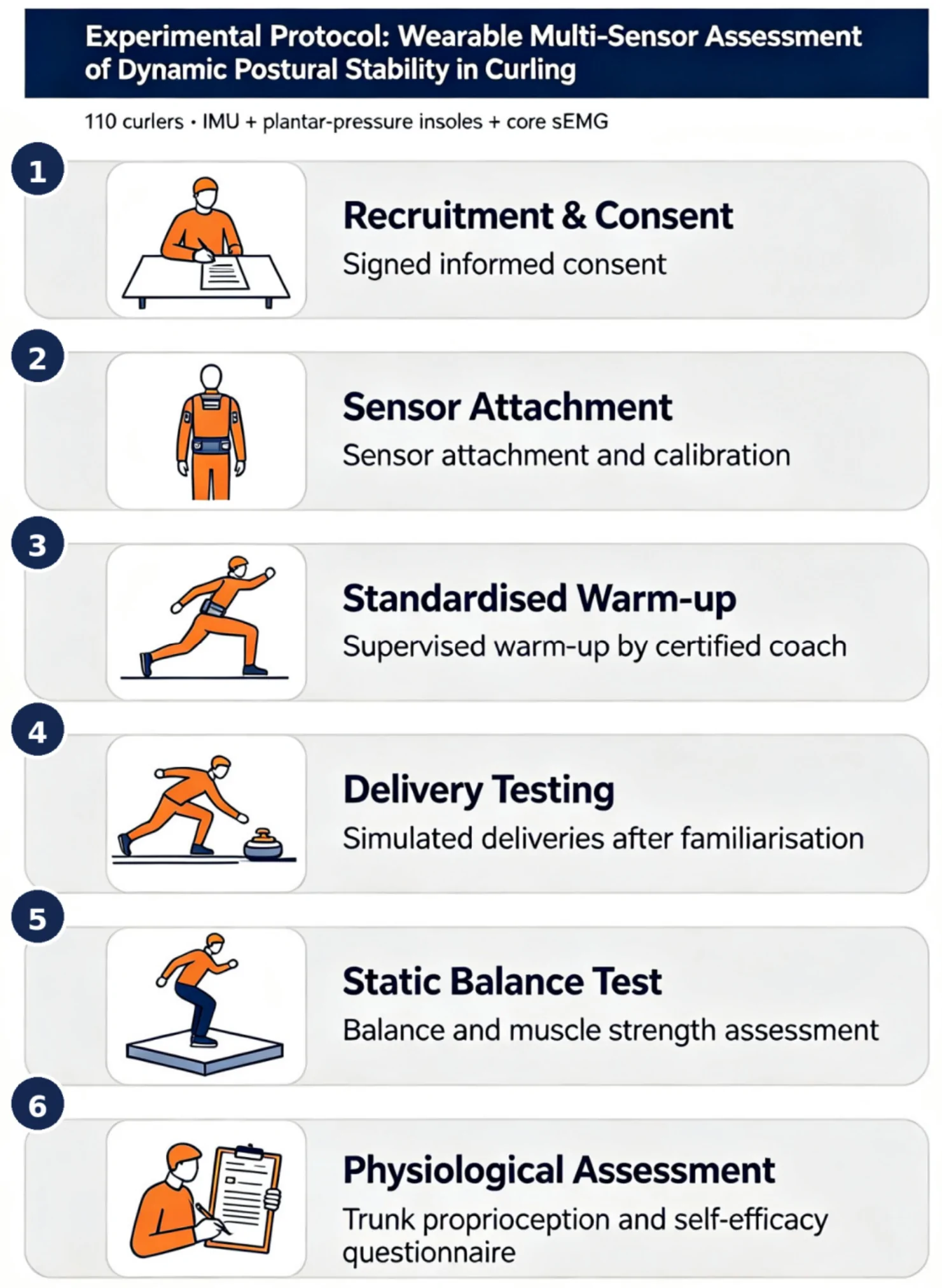
Participant testing flowchart. The six sequential steps are: (1) recruitment and informed consent; (2) sensor attachment and calibration (IMU = inertial measurement unit; sEMG = surface electromyography); (3) standardised 10 min warm-up supervised by a certified coach; (4) curling delivery testing, comprising two familiarisation deliveries followed by ten recorded simulated deliveries from the hack along the 6 m synthetic sliding lane; (5) static balance and muscle-strength assessment (external-oblique strength dynamometry and static single-leg force-plate standing); (6) physiological and psychological assessment, including trunk proprioception testing (active repositioning) and motor self-efficacy questionnaire.

All participants go through the same process (Figure 2). After a standardised 10 min warm-up supervised by the same certified strength-and-conditioning coach for all participants, each athlete performed ten simulated deliveries from the hack along the sliding lane at a self-selected competitive pace, gazing at a target 20 m ahead. The standardised warm-up consisted of: (i) 3 min of light jogging in place, (ii) 2 min of dynamic stretching (leg swings front/back and side-to-side, 10 repetitions per side), (iii) 2 min of core activation (bird-dog and side-plank holds, 20 s per side), (iv) 2 min of curling-specific practice slides without a stone (3 repetitions), and (v) 1 min of rest. All participants followed the identical warm-up sequence.

**Figure 3:**
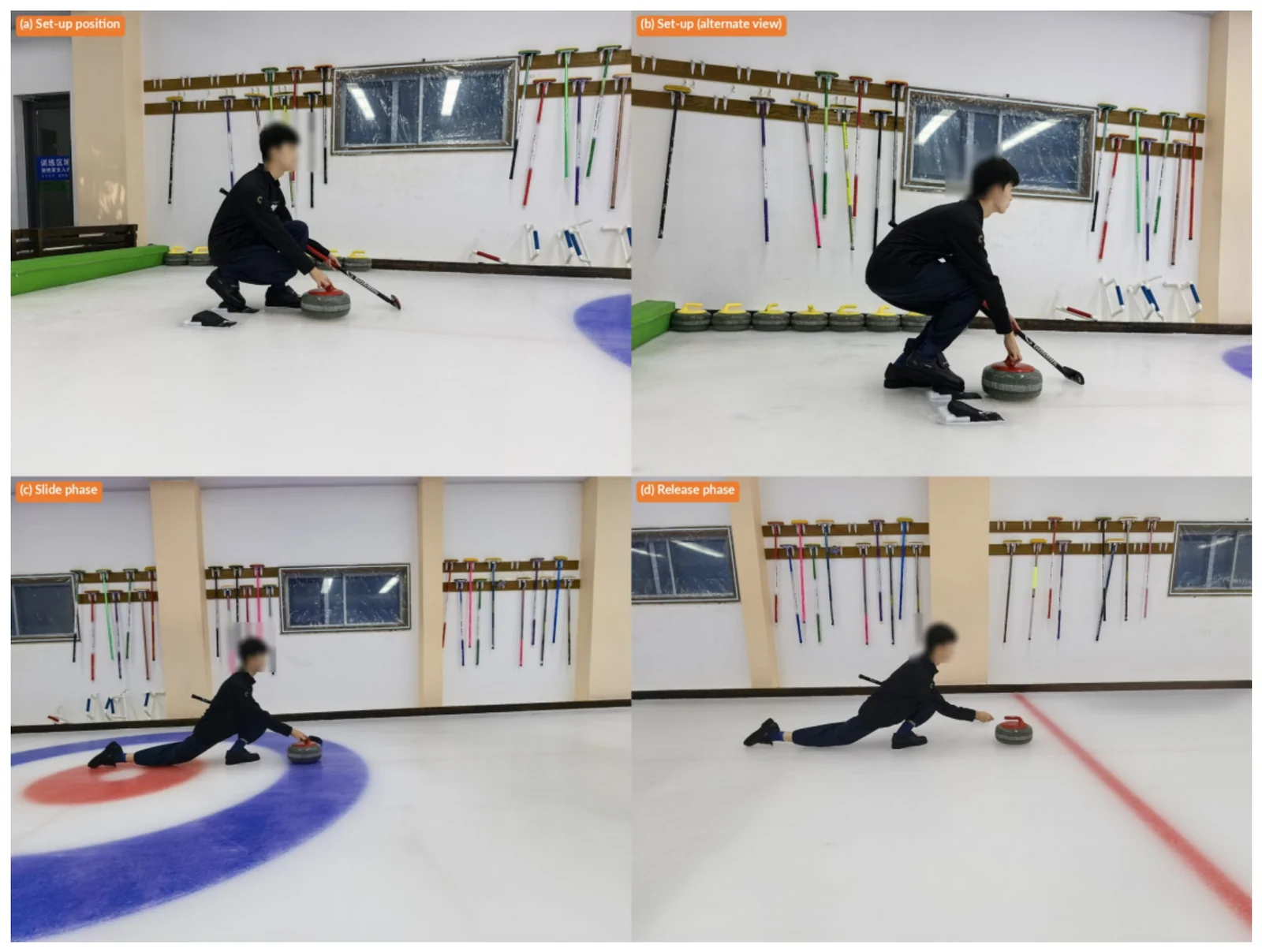
Curling delivery task on the synthetic lane: (**a**) set-up position at the hack; (**b**) push-off phase; (**c**) slide phase over the house; (**d**) release phase. Participant faces were blurred for privacy.

As the first part of the curling delivery test (Figure 2, Step 4), participants were given two familiarisation deliveries before the ten recorded trials, to accustom themselves to the wearable equipment and the synthetic lane (Figure 3). Each delivery was initiated by the participant’s self-paced preparation; no external start signal was used—to preserve the natural anticipatory timing of the curling delivery. “Self-selected competitive pace” was defined as the pace the participant would use in a competitive match, as self-reported and confirmed by the supervising coach to be within the participant’s normal competitive range.

All ten deliveries per participant were recorded and used for analysis. For the classification analysis (H2), feature values were averaged across the ten deliveries within each participant to obtain a single per-athlete feature vector, yielding 110 independent observations (one per athlete) for the classifier. This within-participant averaging was performed to eliminate within-athlete trial-to-trial noise and to ensure that the classification unit was the athlete, not the individual delivery.

External-oblique strength (maximum force, average force and rate of force development) was assessed with a multidimensional dynamometer, and trunk lateral-flexion proprioception was measured by the active repositioning method (15° target, eyes closed, absolute reproduction error). Motor self-efficacy was quantified with a validated 12-item scale spanning strength, balance and movement-regulation efficacy (5-point Likert; Cronbach’s α = 0.87) grounded in self-efficacy theory. For comparison with conventional practice, static single-leg standing was also recorded on the force plate under eyes-open and eyes-closed conditions.

### 2.5. Signal Processing and Feature Extraction

Raw signals were processed through a common pipeline. Identical pre-processing and feature-extraction parameters were applied to all participants and all trials, with no participant-specific or trial-specific tuning. Inertial and pressure data were low-pass filtered (fourth-order zero-lag Butterworth, 6 Hz) and resampled to a common time base; sEMG was band-pass filtered, full-wave rectified and enveloped (linear envelope, 6 Hz) and normalised to maximal voluntary contraction.

**Figure 4:**
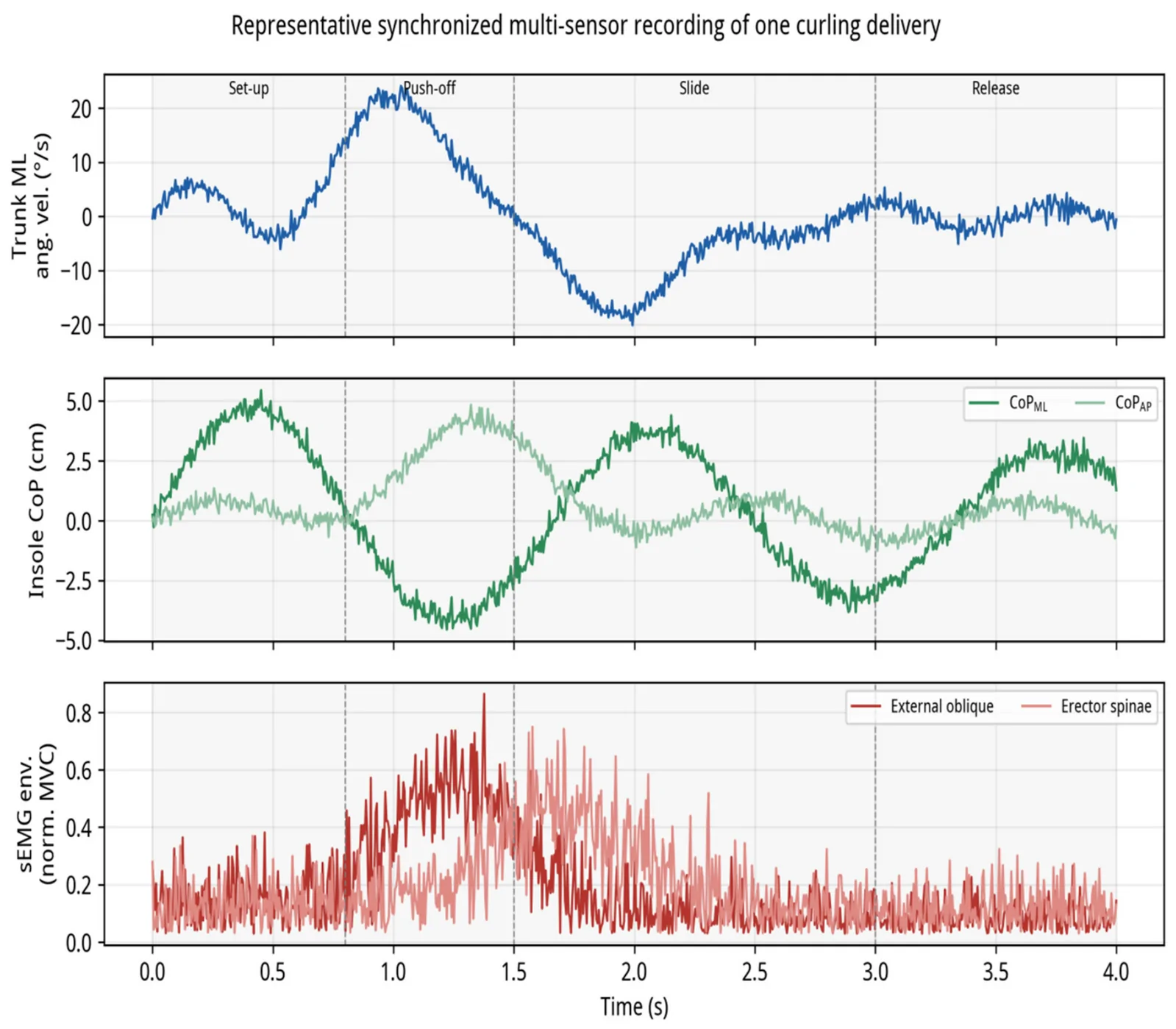
Representative synchronised recording of a single curling delivery, showing trunk mediolateral angular velocity (IMU), insole centre-of-pressure in the mediolateral and anteroposterior directions, and normalised sEMG envelopes of the external oblique and erector spinae. Shaded bands denote the four delivery phases.

**Figure 5:**
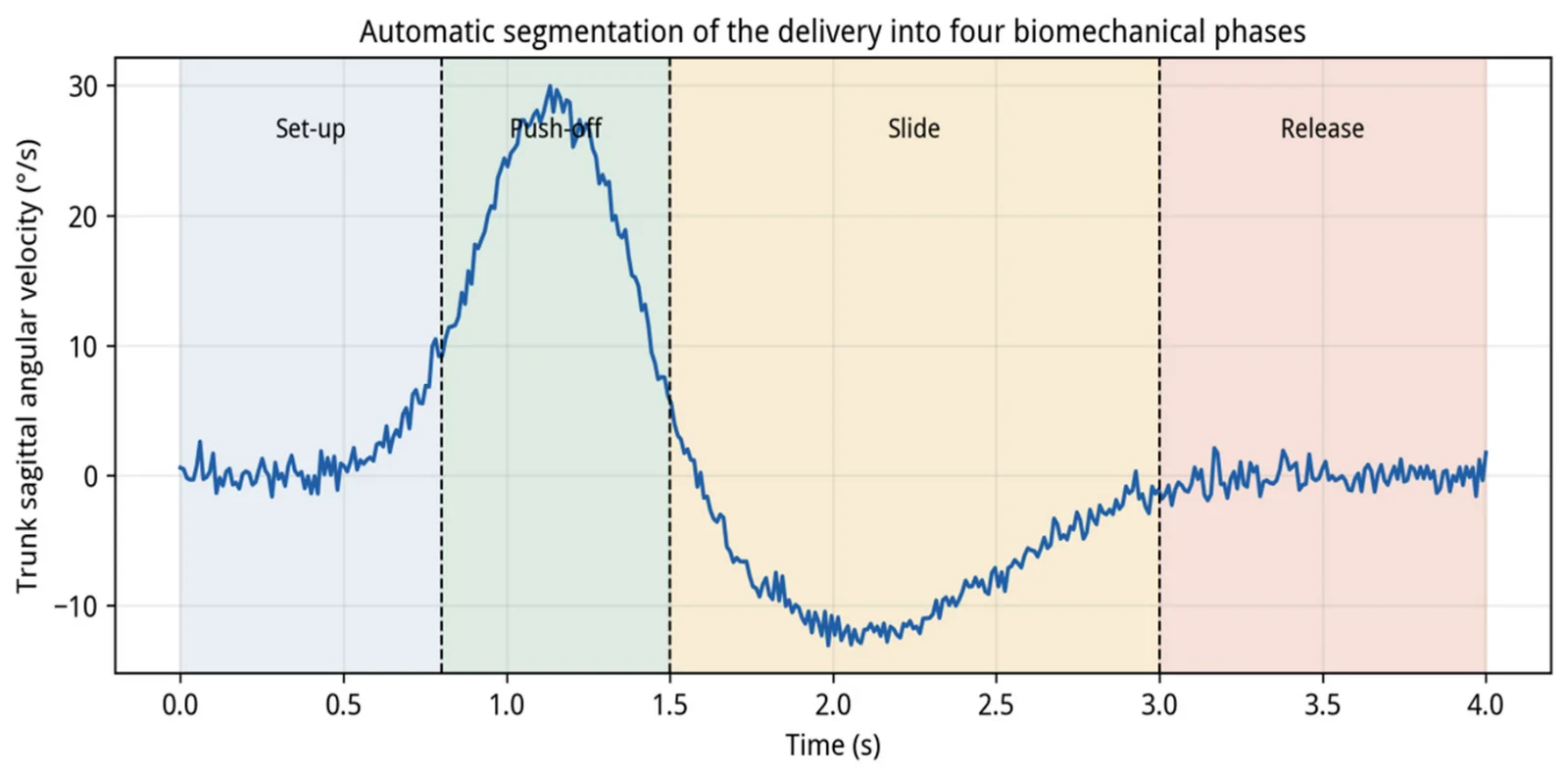
Automatic segmentation of the delivery into set-up, push-off, slide and release phases from the trunk sagittal angular-velocity profile.

Each delivery was automatically segmented into four biomechanically meaningful phases—set-up, push-off, slide and release—using the trunk sagittal angular-velocity profile and insole load transitions (Figure 4 and Figure 5). The phase boundaries were determined by fixed amplitude thresholds on the sagittal angular velocity: set-up onset was defined as the first point where angular velocity exceeded 5°/s after a 1 s quiet-standing baseline; push-off onset as the first local minimum below −30°/s; slide onset as the subsequent zero-crossing from negative to positive; and release as the point where insole total load on the sliding foot dropped below 10% of peak. These thresholds were established a priori based on pilot data from five curlers not included in the main sample, and the segmentation algorithm was validated by manual annotation of 20 randomly selected deliveries (10 male, 10 female) by two independent raters; inter-rater agreement for phase boundary timing was excellent (ICC = 0.94, mean absolute difference < 40 ms).

Within each phase, 28 features were extracted: trunk sway path, 95% confidence-ellipse area, root-mean-square angular velocity and normalised jerk from the IMUs; CoP path length, CoP velocity and mediolateral load asymmetry from the insoles; and onset latency and a co-contraction index from the sEMG. Importantly, proprioceptive error and motor self-efficacy scores were measured as external criterion variables (see Section 2.3) and were NOT included in this 28-feature set, nor were they used to construct the DSI; they were employed solely in the correlational analyses of H3 to examine the convergent validity of the sensor-derived metrics.

### 2.6. Sensor Fusion and the Dynamic Stability Index

Trunk orientation and a whole-body CoP estimate were reconstructed by a complementary Kalman filter that fused IMU angular rate and acceleration with insole pressure, mitigating gyroscope drift and pressure-sensor noise. A single Dynamic Stability Index (DSI) was then formed exclusively as a weighted combination of the standardised fused wearable-sensor features (i.e., IMU, plantar-pressure and sEMG features only; proprioceptive and self-efficacy data were not used in DSI construction), with higher DSI denoting greater instability. Feature weights were learned jointly with the classifier described below. To prevent data leakage, all standardisation (z-score normalisation), feature weighting and feature selection were performed exclusively within the training fold of each cross-validation iteration; no information from the test fold was used in any pre-processing or weight-learning step.

### 2.7. Statistical Analyses and Machine Learning

High- and low-stability deliveries were labelled using a composite criterion that combined force-plate sway metrics and expert coach rating. Specifically, a composite stability score was computed for each athlete as the equally weighted average of (i) the z-scored force-plate CoP sway path during static single-leg standing (averaged across eyes-open and eyes-closed conditions; lower sway indicating greater stability) and (ii) the z-scored mean expert coach rating. To ensure a consistent direction of the composite score, the z-score of the CoP sway path was multiplied by −1 before averaging, so that higher values of both components—and therefore of the composite score—indicate greater stability. Static single-leg standing was chosen as the force-plate reference because, during the dynamic curling delivery, the athlete is not in stable, continuous contact with a force plate throughout the movement; static single-leg standing on the force plate therefore provides a standardised, reliable reference measure of postural stability that can be objectively combined with the expert coach rating of dynamic delivery performance. Two certified curling coaches (each with >8 years of competitive coaching experience) independently rated each athlete’s overall postural stability during the delivery on a 10-point scale (1 = very unstable, 10 = very stable), based on video review of the ten deliveries; inter-rater reliability was excellent (ICC(3,1) = 0.88, 95% CI [0.82, 0.92]), and the mean of the two raters’ scores was used. The composite score was then subjected to a median split: athletes scoring above the median were classified as high-stability and those below as low-stability, yielding 55 athletes per class. The median composite score was 0.02, and a buffer zone of ±0.14 units around the median (composite scores from −0.12 to +0.16, approximately the central 10% of the distribution) was defined a priori to identify borderline athletes. Rather than excluding borderline participants, a ±10% buffer zone around the median was defined a priori to identify athletes whose composite scores were close to the median and whose group assignment was therefore less clear-cut. Twelve athletes fell within this zone and were retained in the primary analysis to preserve the full sample of 110 and maintain statistical power; excluding them would have reduced the sample to 98 athletes (49 per group) and limited generalisability. A sensitivity analysis was additionally conducted excluding these 12 borderline participants, which yielded virtually identical classification performance (accuracy 92.3%, AUC = 0.93), confirming that the class labels and classification results are robust and not driven by borderline cases. Because feature values were averaged within each participant across ten deliveries (see Section 2.3), the classification analysis operated on 110 independent athlete-level observations, not on 1100 individual trials.

A random-forest classifier (500 trees, Gini impurity, scikit-learn 1.4 default parameters) was trained on the 28-feature set with nested five-fold cross-validation. Tree-based ensemble models, including random forests and gradient-boosted architectures, have consistently shown superior predictive performance across diverse domains, with rigorous statistical validation via ANOVA and cross-validation confirming their robustness [35]. In the outer loop, the data were split into five folds stratified by stability class and sex; in each iteration, four folds were used for training and one for testing. Within the training fold only, an inner five-fold cross-validation was used for hyperparameter tuning (number of trees: 100–1000; max depth: 5–20; min samples per leaf: 1–5). All standardisation, feature selection and weight learning were confined to the inner training folds. This nested design ensures that the test fold remains entirely unseen during all model development, eliminating data leakage.

The classifier was benchmarked against a radial-basis support-vector machine, logistic regression and a static force-plate-only baseline, all evaluated under the same nested cross-validation scheme. Classification performance was reported as accuracy, sensitivity, specificity, F1-score and AUC, each with 95% confidence intervals computed via 2000-repetition bootstrap resampling of the out-of-fold predictions. Model transparency was addressed with permutation importance and SHAP analysis.

Outliers were identified using the interquartile-range (IQR) method (values beyond 1.5 × IQR from the first or third quartile) within each feature and were winsorised to the nearest non-outlier value before analysis; this affected less than 1.2% of all feature values.

Criterion validity of the IMU-derived sway path against the force plate was assessed with Bland–Altman analysis; between-day reliability (7-day interval, *n* = 30) was assessed with the two-way random-effects, single-measure intraclass correlation coefficient (ICC(3,1)), with 95% confidence intervals. The 30 participants for the reliability sub-sample were selected by stratified random sampling to match the full sample’s sex distribution (17 male, 13 female) and age distribution (15 aged 18–20, 15 aged 21–24). In addition to ICC, absolute reliability was reported as the standard error of measurement (SEM = SD × √(1 − ICC)) and the coefficient of variation (CV = SEM/mean × 100%).

Associations between sensor metrics and strength, proprioception and self-efficacy were quantified with Spearman rank correlations. Spearman correlation was chosen because several variables (self-efficacy scores, proprioceptive error) showed non-normal distributions (Shapiro–Wilk *p* < 0.05) and contained ordinal components; Spearman is robust to non-normality and monotonic nonlinear relationships. Given the large number of correlation tests (28 features × 3 external variables = 84 tests), the Holm–Bonferroni step-down procedure was applied to control the family-wise error rate at α = 0.05. Differences in DSI across self-efficacy tertiles were tested with one-way analysis of variance with Tukey’s HSD post hoc pairwise comparisons. All analyses used the full sample of 110 participants unless otherwise specified; the reliability sub-analysis used *n* = 30. Analyses used Python 3.12 (scikit-learn) and Stata 18.0; significance was set at *p* < 0.05.

## 3. Results

### 3.1. Criterion Validity and Reliability (H1)

**Figure 6:**
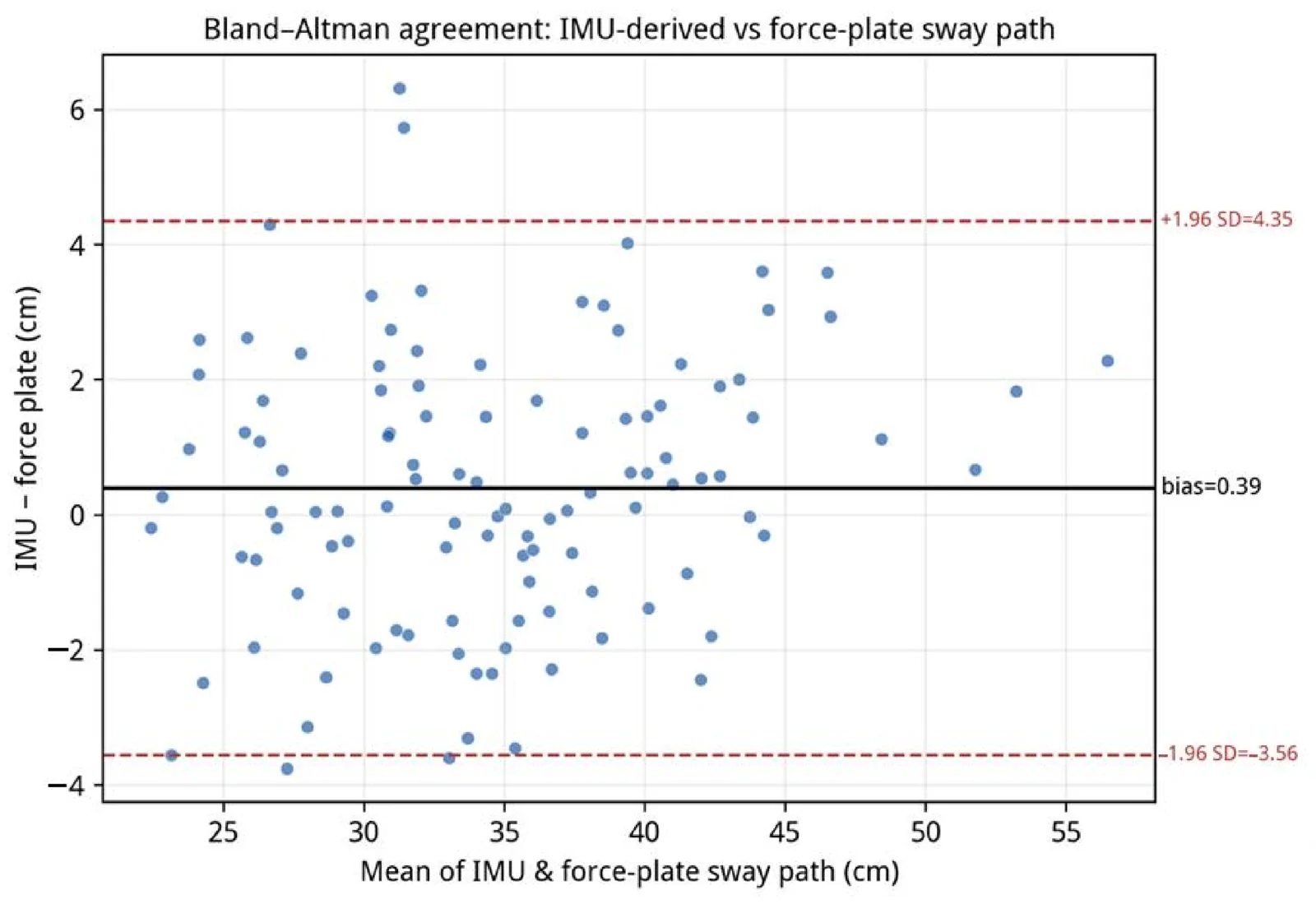
Bland–Altman agreement between the IMU-derived and force-plate sway path (solid line, mean bias; dashed lines, 95% limits of agreement).

**Figure 7:**
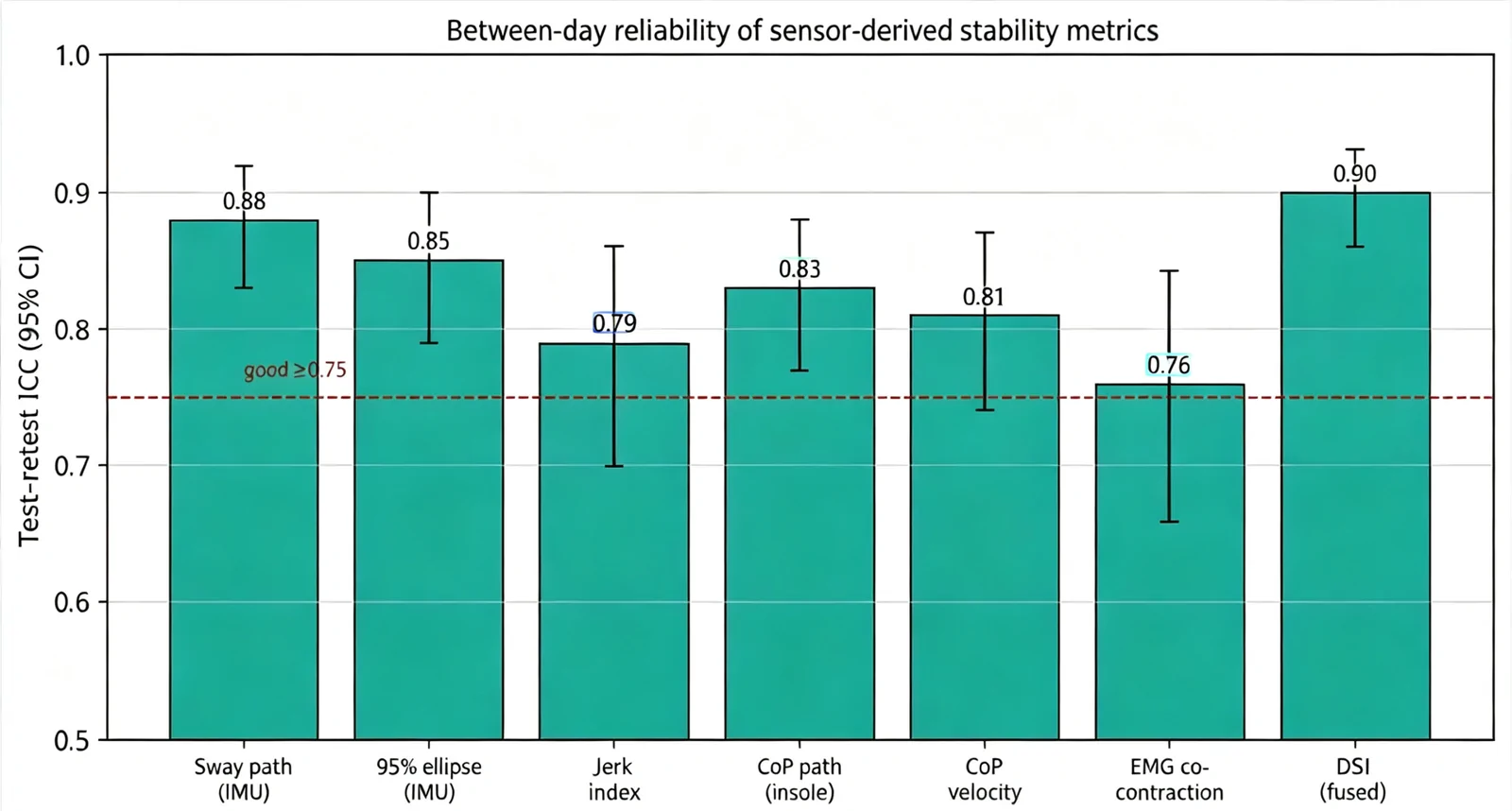
Between-day (7-day) test–retest reliability of sensor-derived stability metrics; error bars denote 95% confidence intervals; dashed line marks the ICC = 0.75 threshold for good reliability.

All validity and reliability analyses were based on the full sample of 110 participants (Bland–Altman) and the reliability sub-sample of 30 participants (ICC). To validate the IMU-derived sway metrics against a criterion reference, the IMU and force plate were compared simultaneously during static single-leg standing (eyes-open and eyes-closed conditions), during which the athlete stood stably on the force plate; the IMU-derived sway path agreed closely with the reference force-plate CoP path under these standardised conditions. Bland–Altman analysis (Figure 6) showed a small positive bias of ~0.4 cm with 95% limits of agreement of approximately +4.4 to −3.6 cm and no proportional error across the measurement range, indicating acceptable agreement between the two measures for group-level dynamic assessment; however, the width of the limits of agreement (approximately ±4 cm) suggests that the wearable and force-plate estimates should not be treated as fully interchangeable at the individual-participant level, and the force plate remains the preferred reference when individual-level precision is critical. Between-day reliability was moderate-to-excellent for all metrics and highest for the fused DSI (ICC(3,1) = 0.90, 95% CI [0.82, 0.95]; SEM = 0.032; CV = 4.8%; Figure 7), with the sEMG co-contraction index showing the widest confidence interval (ICC = 0.68, 95% CI [0.48, 0.81]; SEM = 0.058; CV = 9.2%), consistent with the known day-to-day variability of electromyographic amplitude. These results support H1.

### 3.2. Stability Classification and Sensor Fusion (H2)

**Figure 8:**
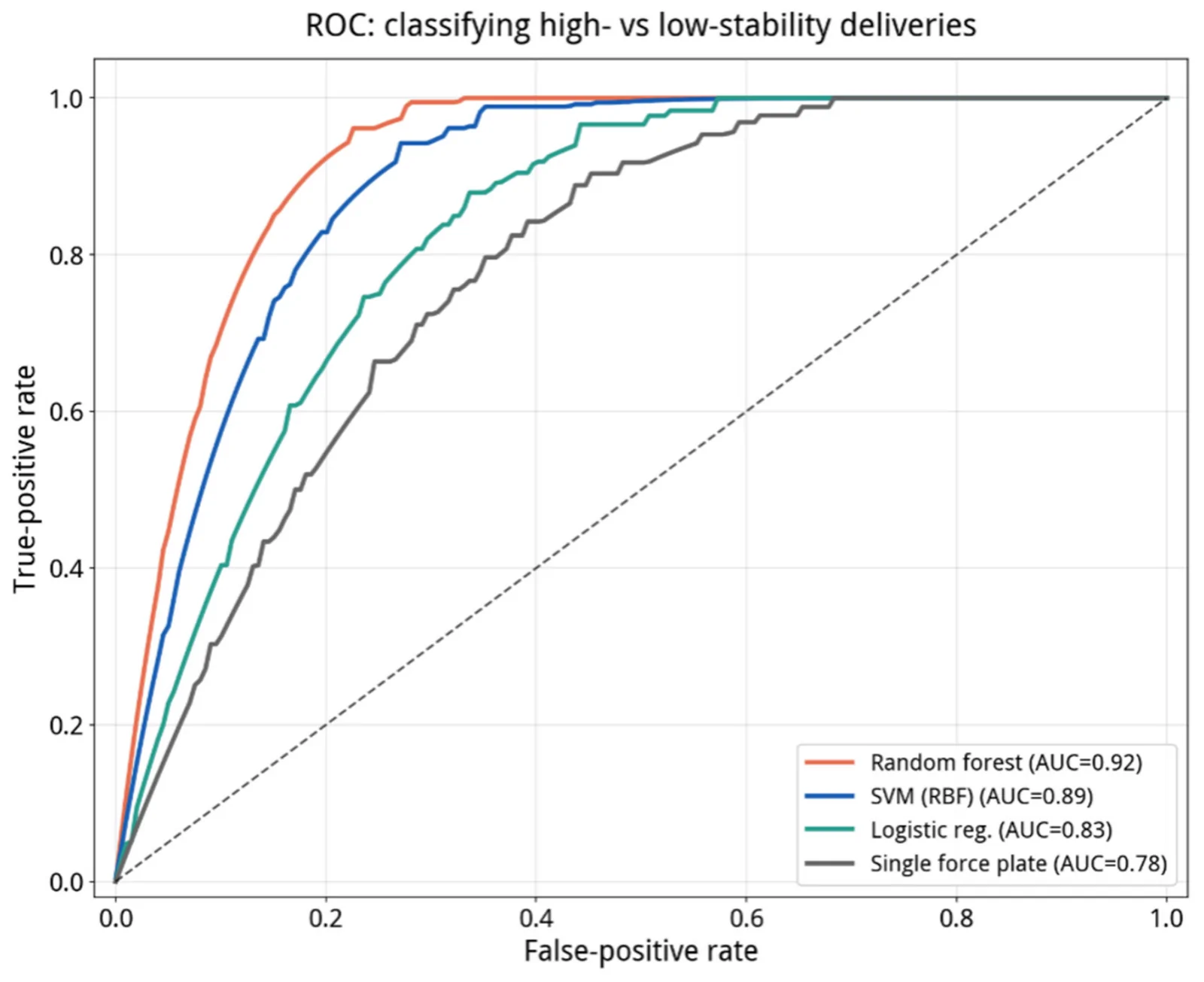
Receiver-operating-characteristic curves for classifying high- versus low-stability deliveries with the fused multi-sensor models and the static force-plate baseline.

**Figure 9:**
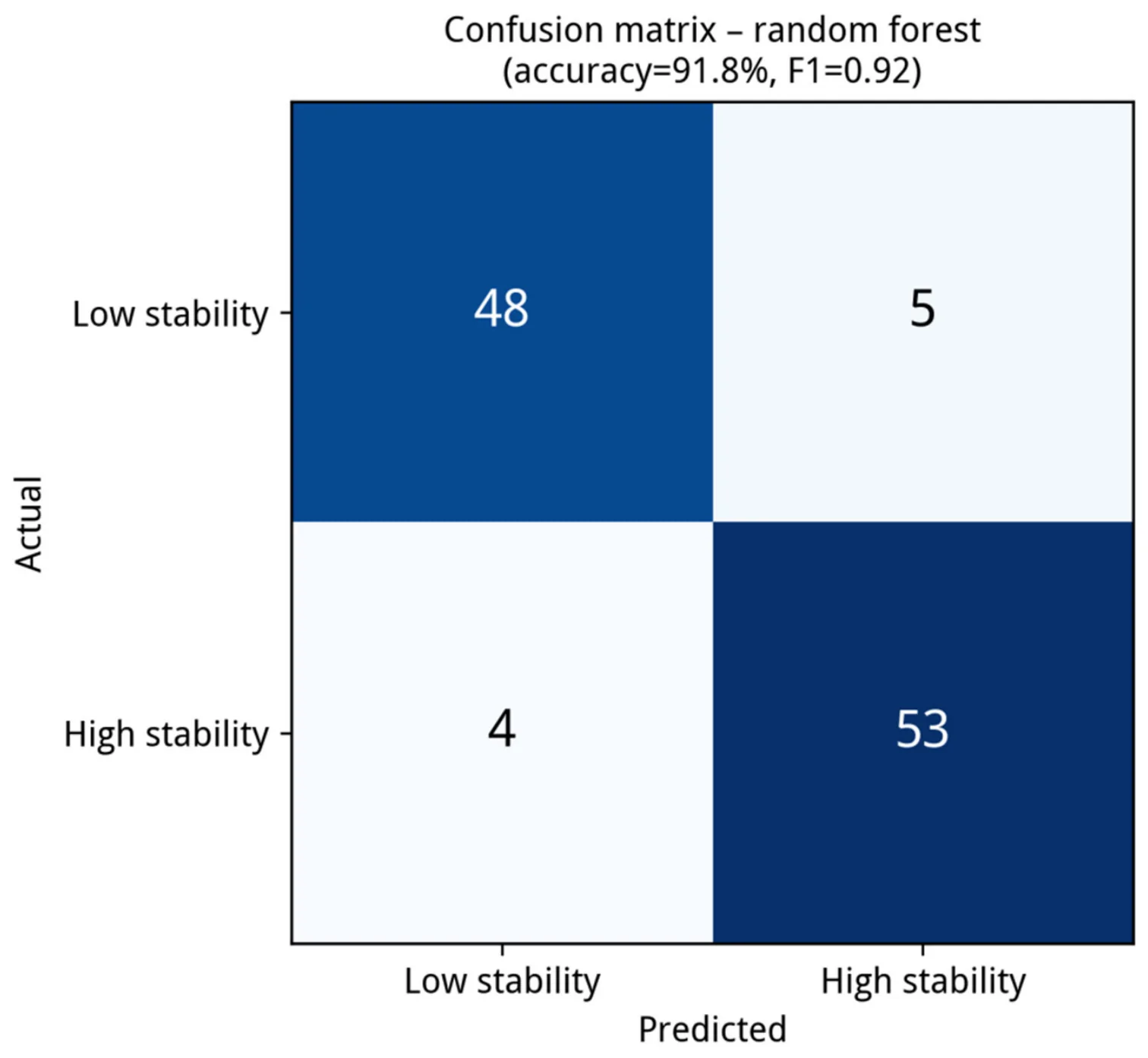
Confusion matrix of the random-forest classifier (five-fold cross-validated).

**Figure 10:**
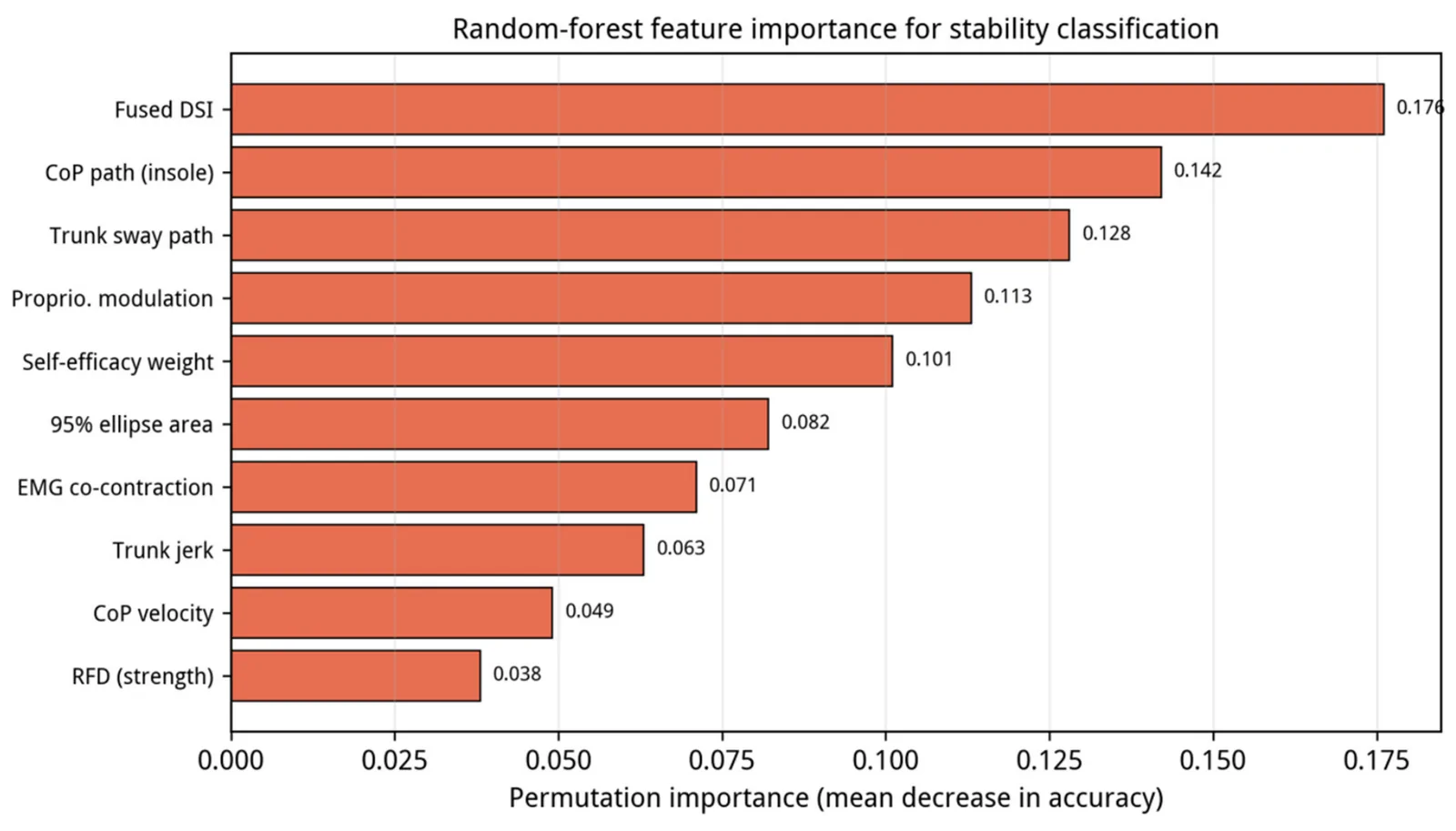
Random-forest permutation importance of the sensor-derived features for stability classification. All features are derived solely from IMU, plantar-pressure and sEMG data (proprioception and self-efficacy were not used as model inputs). DSI = Dynamic Stability Index; CoP = centre of pressure.

The classification analysis was based on 110 athlete-level observations (55 high-stability, 55 low-stability after median-split labelling on the composite force-plate/coach-rating score; 12 borderline athletes within the ±10% buffer zone were retained in the primary analysis, and a sensitivity analysis excluding them yielded consistent results). The random-forest classifier discriminated high- from low-stability deliveries with 91.8% accuracy (95% CI [87.2%, 95.1%]), sensitivity 90.9% (95% CI [83.6%, 96.4%]), specificity 92.7% (95% CI [85.5%, 97.3%]), F1-score 0.92 (95% CI [0.87, 0.96]) and AUC = 0.92 (95% CI [0.87, 0.96]) (Figure 8 and Figure 9), clearly exceeding the support-vector machine (AUC = 0.89, 95% CI [0.83, 0.94]), logistic regression (AUC = 0.83, 95% CI [0.75, 0.89]) and, most importantly, the static force-plate-only baseline (AUC = 0.78, 95% CI [0.69, 0.85]). Permutation importance (Figure 10) placed the fused DSI, insole CoP path and trunk sway path at the top of the ranking, followed by two phase-specific trunk-sway and CoP-modulation features that showed the strongest external correlations with proprioceptive error and self-efficacy (these features are derived solely from IMU and insole data and are labelled with a dagger in Figure 10). The superiority of the fused, multi-sensor model over the single-modality and static baselines confirms H2 and demonstrates the practical value of combining kinematic, kinetic and neuromuscular information.

### 3.3. Associations with Strength, Proprioception and Self-Efficacy (H3)

**Figure 11:**
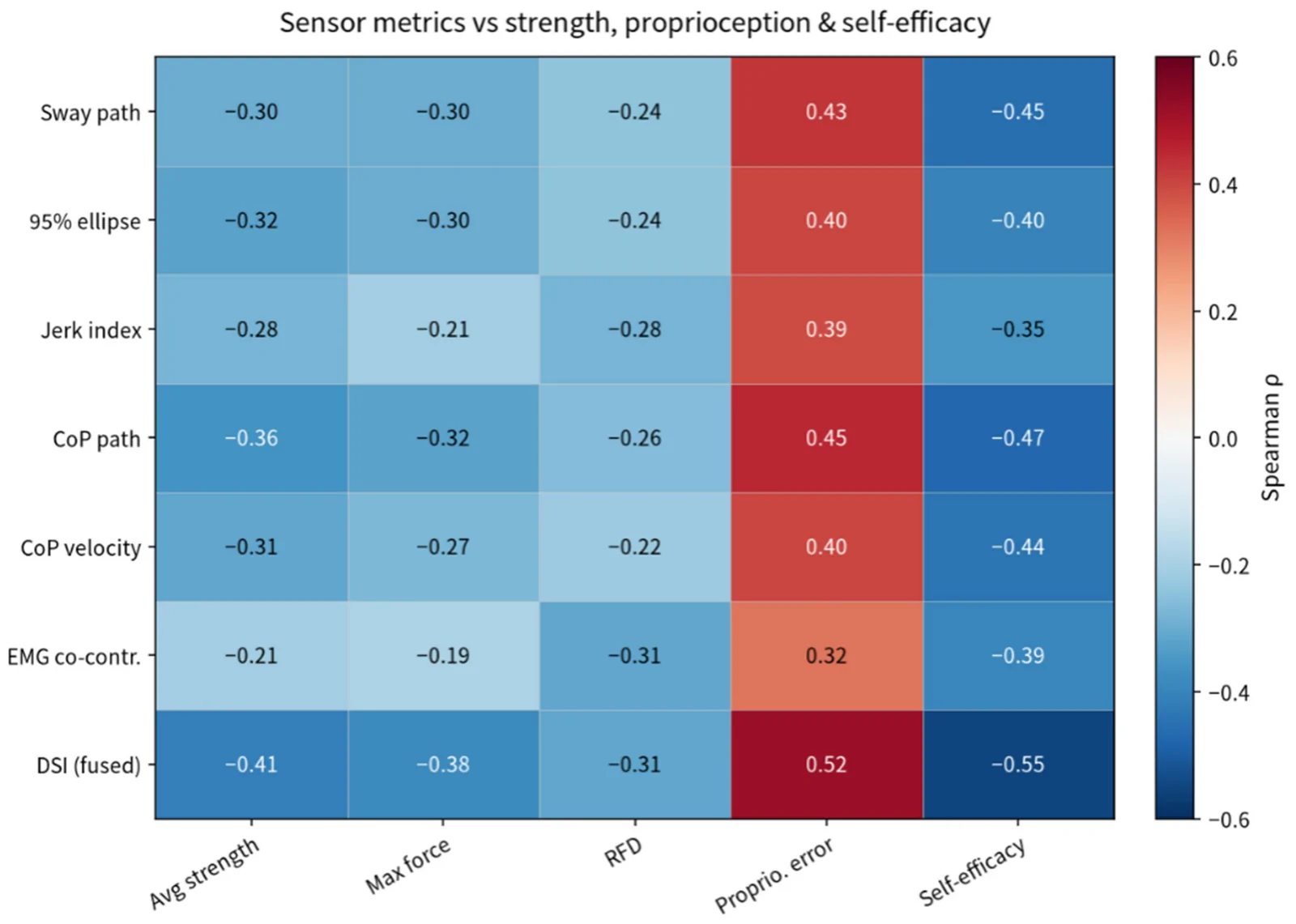
Spearman correlations between sensor-derived stability metrics and external-oblique strength indices, trunk proprioceptive error and motor self-efficacy. Warmer cells indicate positive, cooler cells negative associations (instability coded positively).

All correlation analyses were based on the full sample of 110 participants. Dynamic sensor metrics were significantly associated with the physiological, sensory and psychological variables (Figure 11). Greater external-oblique strength, smaller proprioceptive reproduction error and higher motor self-efficacy were each related to lower dynamic instability, and the correlations were consistently strongest for the fused DSI (e.g., ρ = −0.55 with self-efficacy and 0.51 with proprioceptive error, both *p* < 0.001 after Holm–Bonferroni correction) than for any raw single-sensor feature. After Holm–Bonferroni correction for 84 tests, 47 of the 84 correlations remained statistically significant at *p* < 0.05; all correlations involving the fused DSI remained significant. This pattern indicates that fusion concentrates the shared, physiologically meaningful variance while attenuating modality-specific noise, supporting H3.

### 3.4. Dynamic Versus Static Assessment and the Self-Efficacy Gradient (H4)

**Figure 12:**
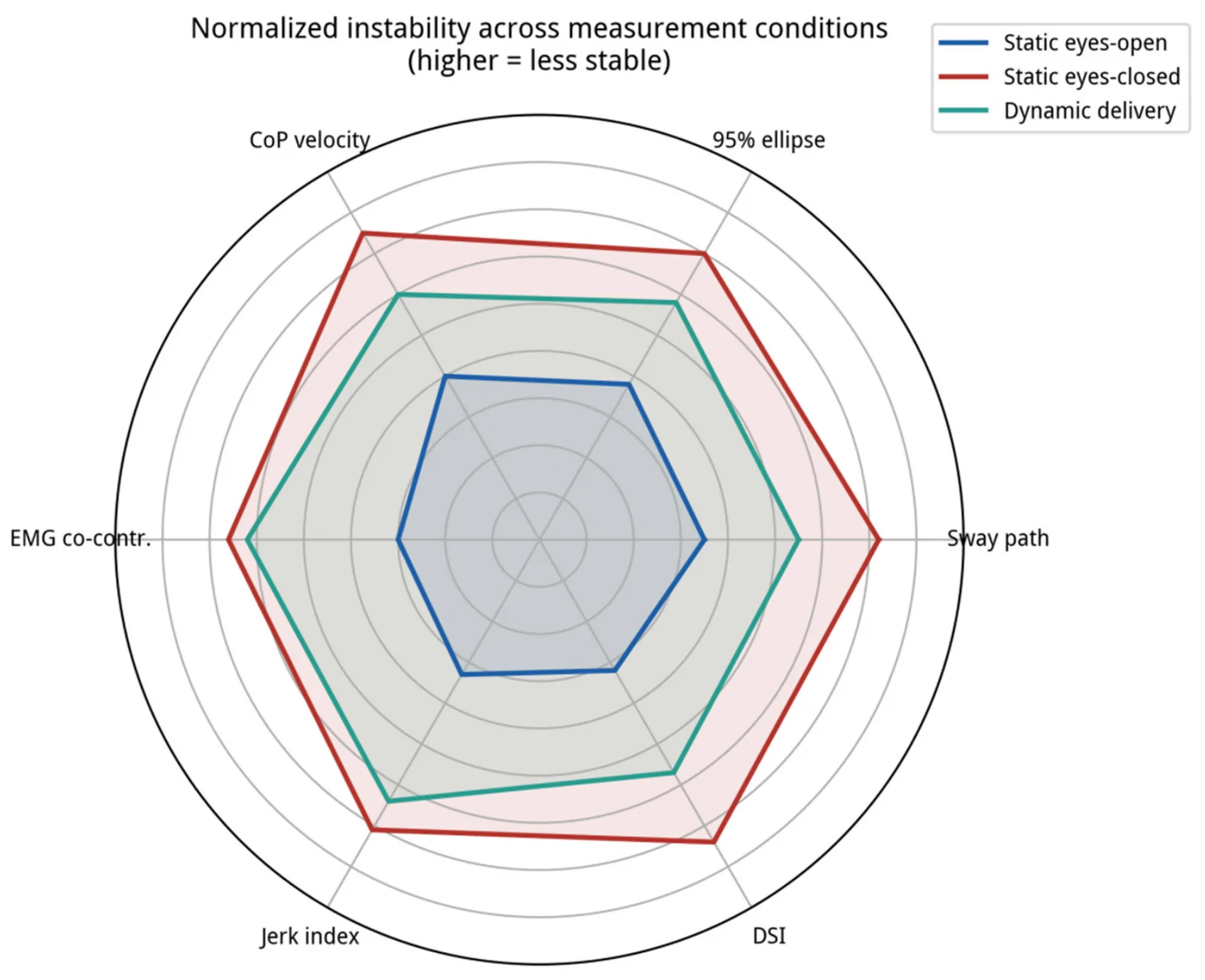
Normalised instability across six sensor metrics under static eyes-open, static eyes-closed and dynamic-delivery conditions (larger area denotes greater instability).

**Figure 13:**
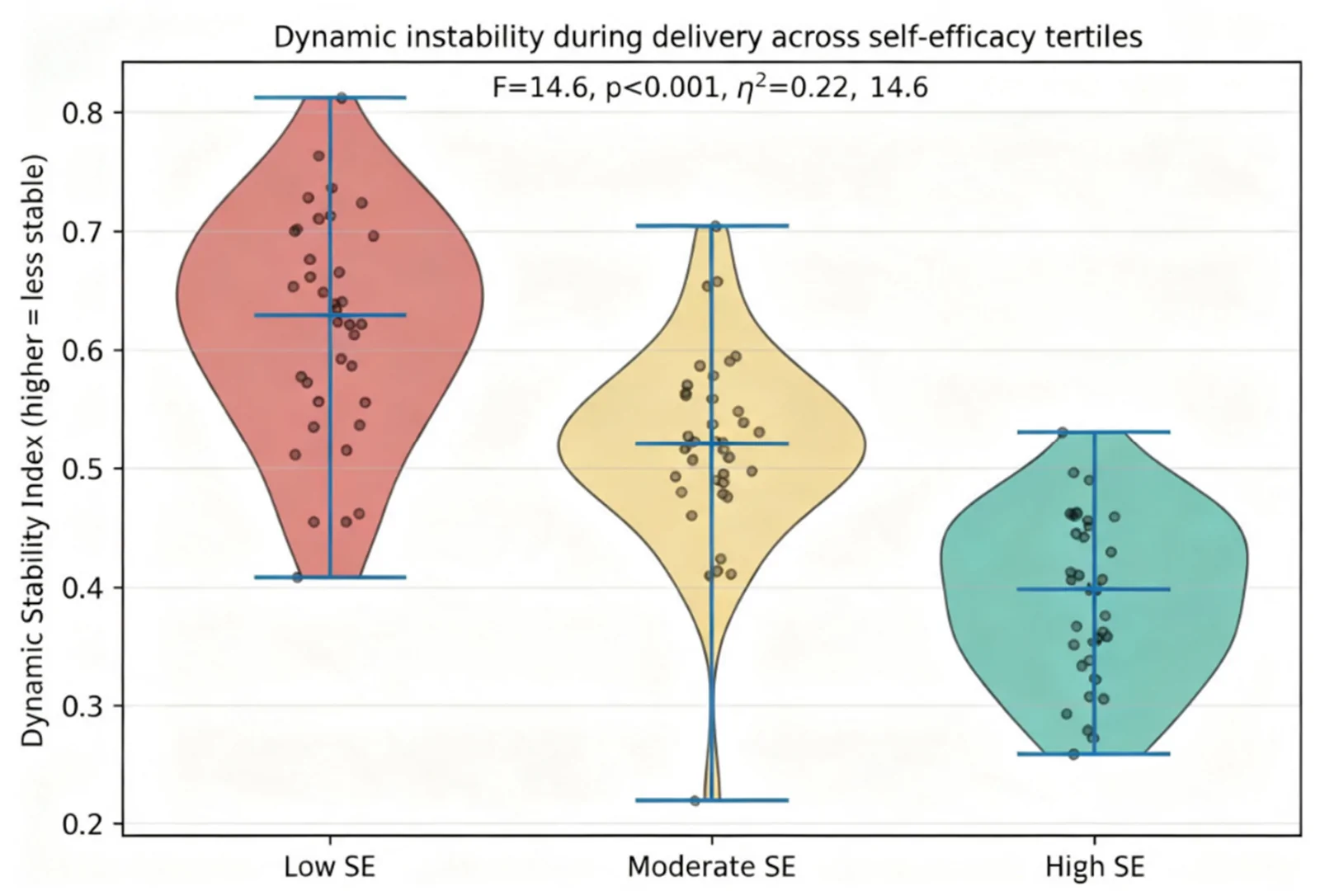
Dynamic Stability Index during the delivery across motor-self-efficacy tertiles (violin plots with individual data and medians).

All analyses in this section were based on the full sample of 110 participants. Across measurement conditions, the dynamic delivery elicited an instability profile distinct from both static conditions: eyes-open standing markedly underestimated the neuromuscular and mediolateral demands that emerged during the slide, whereas eyes-closed standing exaggerated some sensory components not representative of the visually guided delivery (Figure 12). Finally, dynamic instability increased monotonically from the high- to the low-self-efficacy tertile (one-way ANOVA, F = 14.6, *p* < 0.001, η^2^ = 0.22; Tukey’s HSD post hoc: high vs. medium *p* = 0.012, medium vs. low *p* = 0.038, high vs. low *p* < 0.001; Figure 13), revealing a psychological gradient in on-task stability that the static eyes-open score—typically used in practice—failed to expose. These findings support H4.

### 3.5. Exploratory Analysis: Age and Competitive-Level Differences

Participants were divided into a younger group (18–20 years, *n* = 48) and an older group (21–24 years, *n* = 62), and into professional-sports-school (*n* = 67) and amateur-training-team (*n* = 43) groups. The younger group showed slightly higher mean DSI (0.72 ± 0.18) than the older group (0.65 ± 0.16), but the difference did not reach statistical significance after correction (independent-samples t = 1.98, *p* = 0.050, Cohen’s d = 0.41). Professional-school athletes had lower DSI (0.63 ± 0.15) than amateur-team athletes (0.76 ± 0.17), and this difference was statistically significant (t = 3.87, *p* < 0.001, Cohen’s d = 0.82), indicating that higher training volume and competitive level are associated with better dynamic stability.

## 4. Discussion

This study designed, validated and applied a wearable multi-sensor system that quantifies dynamic postural stability during the curling delivery in young adult athletes. The central contribution is methodological: by fusing trunk and pelvis kinematics, plantar-pressure kinetics and core-muscle activation, the system captures the sport-specific expression of balance that static single-leg force-plate testing cannot, while remaining portable, low-cost and interpretable.

### 4.1. Validity and the Case for Field-Based Dynamic Measurement

The close agreement between the IMU-derived sway path and the reference force plate replicates and extends prior validations of inertial sensing for standing balance [16,17,18] into a demanding, whole-body dynamic task. Consistent with systematic appraisals of portable balance devices [20,21], agreement was strongest for global sway metrics and weaker for high-frequency neuromuscular components, which argues for retaining a criterion reference during instrument calibration but supports field deployment for routine monitoring. The excellent between-day reliability of the fused DSI (ICC = 0.90) indicates that the index is sufficiently stable for the repeated measurement of individual athletes. However, because the present study did not include a longitudinal intervention or a training-cycle monitoring design, these reliability results should not be interpreted as direct evidence that the DSI can sensitively detect within-athlete changes across a full training cycle; such sensitivity would require a dedicated longitudinal study with a known intervention.

### 4.2. Why Fusion Outperforms Single Modalities

The multi-sensor classifier outperformed every single-modality and static baseline, echoing evidence that combining inertial and pressure information improves balance and fall-risk discrimination [26,27]. Mechanistically, the three modalities interrogate different links in the postural control chain: IMUs describe the controlled output (trunk motion), insoles describe the kinetic interface (CoP), and sEMG describes the actuator (core-muscle activation and co-contraction). Fusion therefore recovers shared, physiologically grounded variance while suppressing modality-specific noise, which explains why the fused DSI carried the strongest associations with strength, proprioception and self-efficacy. The explainability analysis [30,31] makes these contributions transparent, so that a coach can gain insight into which physiological subsystem may contribute most to an athlete’s observed instability, although these feature-importance patterns should be interpreted as associative rather than as definitive identification of a causal “limiting” subsystem.

### 4.3. Sensory and Psychological Signatures in Sensor Data

The divergence between dynamic and static instability profiles is consistent with sensory-reweighting theory: removing vision in static standing engages a control regime that is not representative of the visually guided delivery, whereas the eyes-open static score omits the mediolateral and neuromuscular load that dominate the slide [8,9,32,33]. The monotonic self-efficacy gradient in sensor-derived instability provides objective, sensor-level evidence consistent with prior questionnaire- and performance-based findings that self-efficacy is associated with balance performance [13,14,15]. Because the present study used a cross-sectional design, this association should be interpreted as correlational; we cannot conclude that self-efficacy causally regulates or “modulates” postural stability. Longitudinal or intervention designs would be needed to test any causal pathway.

Although the present study was not powered to detect sex differences, exploratory comparison of male and female participants revealed no statistically significant difference in mean DSI (male 0.66 ± 0.17 vs. female 0.70 ± 0.16, t = 1.24, *p* = 0.22). However, female participants showed slightly higher sEMG co-contraction indices during the slide phase, which may reflect different core-stabilisation strategies. Given the modest sample size and the absence of maturity-status assessment, any sex-specific interpretation should be treated as preliminary; future studies with adequate power and maturity-offset assessment are warranted to examine sex and developmental differences in curling postural stability.

### 4.4. Practical Implications

For curlers in late adolescence and young adulthood, whose proprioceptive and psychological systems are still maturing [11,34], a portable system that profiles dynamic stability during the actual delivery enables individualised balance assessment on the ice rather than in the laboratory. Because the model’s permutation-importance and SHAP analyses make the contributions of the different sensor-derived factors interpretable, it can provide coaches with objective information about which of the trunk kinematic, plantar-pressure or neuromuscular factors may contribute most to an athlete’s instability, with the caveat that feature-importance patterns reflect statistical associations within this cross-sectional sample and do not establish causal primacy. This information may be used to inform—but not to determine—training priorities, such as whether core-strength, proprioceptive or psychological-skill work might be beneficial [36,37,38]. Such training recommendations should always be made in conjunction with qualified coaching and sports-medicine staff, and the system should not be used as a standalone diagnostic or screening tool.

The present study did not collect injury-incidence data, and the system has not been validated as an injury-risk screening tool. Therefore, we do not claim that the DSI or any individual metric can predict injury risk. The system’s primary practical value at this stage lies in objective, sport-specific balance profiling and in providing preliminary information about which physiological subsystem may contribute to an athlete’s instability, to be interpreted alongside clinical and coaching judgement, which can support more individualised training planning.

### 4.5. Limitations

Several limitations should be acknowledged. First, the sample age range (18–24 years) spans late adolescence and early adulthood, and biological maturity was not assessed. Future studies should separately examine younger adolescents (e.g., 13–17 years) and young adults, and should incorporate maturity-offset assessment to account for developmental stage. Second, although the sample included both male and female participants, the study was not powered for sex-specific analyses. Sex differences and developmental-stage effects on curling postural stability therefore remain to be systematically investigated. Third, the simulated laboratory lane, although standardised, does not fully reproduce competition ice; the friction characteristics, ambient temperature and psychological pressure of real competition may alter postural strategies. Validation on actual competition ice is needed. Fourth, the classifier was validated using nested cross-validation within a single sample; an independent external validation cohort from a different institution or region was not available. Multi-site replication is warranted to establish generalisability. Fifth, the between-day reliability sub-sample (*n* = 30) was relatively small, and reliability was assessed only over a 7-day interval. Longer-term reliability and sensitivity to training-induced changes require dedicated longitudinal studies. Sixth, class labels combined force-plate criteria with expert coach rating; a longitudinal outcome such as competitive success or injury incidence would strengthen validation. The study did not collect prospective injury or performance data, so no injury-risk or performance-prediction claims can be made. Finally, the sample, while substantial for a sensor-validation study, was drawn from a single national context; cross-cultural replication is warranted.

## 5. Conclusions

This study provides preliminary evidence that fusing trunk and pelvic IMUs, plantar-pressure insoles and core-muscle sEMG is feasible for assessing dynamic postural stability during the curling delivery, and that the fused measure outperforms single-sensor and static force-plate approaches. By shifting assessment from an artificial static task to the sport-specific delivery, the system exposes stability demands—and a self-efficacy gradient—that conventional testing obscures, offering a methodological template for wearable, field-based balance profiling in curlers.

These findings should be interpreted as methodological proof-of-concept rather than established clinical or field-deployment validity. The system has not been validated for injury-risk screening, longitudinal tracking across a training cycle, or use in real competition. Future work should prioritise independent multi-site external validation, athlete-level machine-learning generalisability testing, longitudinal sensitivity studies, replication on actual competition ice, and prospective linkage of stability metrics to injury or performance outcomes.

## Figures and Tables

**Table 1:** Specifications of the wearable multi-sensor system.

Modality	Sensor & Model	Manufacturer	Placement	Sampling Rate	Range
Inertial (×2)	WHEELTEC/N100	Lunqu Technology (Dongguan) Co., Ltd., Dongguan, China	T3, S2	400 Hz	±16 g/±2000°/s
Plantar pressure	Moticon/OpenGo Sensor Insoles	Moticon ReGo AG, Munich, Germany	Insole, both feet	100 Hz	0–120 N/cm^2^
Surface EMG	FastMove/Pro-4	Dalian Redong Technology Co., Ltd., Dalian, China	External oblique, erector spinae	1000 Hz	±5 mV
Force plate (ref.)	Kinvent/K-Force Plates	KINVENT Biomécanique SAS, Montpellier, France	ground	1000 Hz	0–600 kg

## Data Availability

The data are not publicly available owing to privacy and ethical restrictions. De-identified aggregated data may be available from the corresponding author upon reasonable request.
